# Risk factors and adverse outcomes of extubation failure in preterm infants ≤32 weeks with neonatal respiratory distress syndrome

**DOI:** 10.3389/fped.2025.1555521

**Published:** 2025-07-10

**Authors:** Chen Zhu, Zhengli Wang, Kaizhen Liu, Jiacheng Li, Wenyan Tang, Yuan Shi, Qingxiong Zhu

**Affiliations:** ^1^ Department of Neonatology, Jiangxi Maternal and Child Health Hospital, Nanchang, China; ^2^ Department of Neonatology, Jiangxi Hospital Affiliated to Children's Hospital of Chongqing Medical University, Nanchang, China; ^3^ Department of Neonatology, Children's Hospital of Chongqing Medical University, Chongqing, China

**Keywords:** respiratory distress syndrome, extubation failure, preterm infants, intubation, mechanical ventilation

## Abstract

**Objective:**

Invasive mechanical ventilation (IMV) is a critical intervention for neonatal respiratory distress syndrome (NRDS). However, the high incidence of extubation failure and its adverse impact on preterm outcomes make the optimal timing of extubation a key clinical concern. This study aimed to identify risk factors for initial IMV extubation failure and analyze associated adverse outcomes in neonates ≤32 weeks’ gestation with NRDS, to provide evidence-based guidance for clinical decision-making.

**Method:**

A retrospective cohort study was conducted in the neonatal ICU (NICU) of Jiangxi Maternal and Child Health Hospital from January 2021 to May 2024, including neonates ≤32 weeks with NRDS who are required to receive IMV within 72 h postnatal. Patients were stratified into a success group (*n* = 228) and a failure (*n* = 62) group based on whether reintubation was required within 72 h post-extubation. Multivariable logistic regression and nomogram modeling were employed to analyze independent risk factors.

**Results:**

A total of 290 cases were included, comprising 228 in the successful extubation group and 62 in the failed extubation group, yielding an extubation failure rate of 21.4%. Univariate analysis revealed that the extubation failure group had significantly lower gestational age, birth weight, weight at extubation, and initial serum albumin levels (*p* < 0.05) but higher Day 1 fluid intake, fraction of inspired oxygen (FiO₂) before extubation, incidence of patent ductus arteriosus (PDA) >1.5 mm, and Grade 3 or higher intraventricular hemorrhage (IVH) (*p* < 0.05). Additionally, maternal *Ureaplasma urealyticum* (UU) infection and placental abruption were more prevalent in the extubation failure group (*p* < 0.05). Multivariate logistic regression identified maternal UU infection, placental abruption, lower weight at extubation, higher FiO₂, Grade 3 or higher IVH, and PDA >1.5 mm as independent risk factors for extubation failure (*p* < 0.05). A nomogram model incorporating these six factors demonstrated a sensitivity of 91% and a specificity of 52% for predicting extubation failure, with an area under the curve (AUC) of 0.77. The extubation failure group had higher incidences of atelectasis and bronchopulmonary dysplasia (BPD) and required longer IMV duration during hospitalization (*p* < 0.05).

**Conclusion:**

Lower body weight at extubation, higher FiO₂, patent ductus arteriosus (PDA >1.5 mm), Grade 3 or higher intracranial hemorrhage, maternal *Ureaplasma urealyticum* infection, and placental abruption during pregnancy are independent risk factors for the failure of the first IMV extubation in neonates ≤32 weeks gestational age with NRDS. Extubation failure significantly increases the risk of atelectasis and BPD and prolongs the duration of invasive ventilatory support.

## Introduction

Neonatal respiratory distress syndrome (NRDS) is a common respiratory disorder in preterm infants, with incidence inversely correlated with gestational age (1). It results from primary or secondary deficiency of pulmonary surfactants (PS) and is clinically manifested as grunting, tachypnea, cyanosis, and in severe cases, apnea, shallow breathing, and hypotonia (2). Current international NRDS management aims to maximize survival while minimizing complications such as air leaks and bronchopulmonary dysplasia (BPD) (3). Recent advances in non-invasive ventilation and antenatal corticosteroids have improved preterm outcomes, but invasive mechanical ventilation (IMV) remains vital for critically ill neonates. Its objectives include promoting gas exchange, correcting respiratory acidosis, relieving CO₂ retention, and reducing respiratory muscle workload (4). However, prolonged ventilation is associated with adverse outcomes such as ventilator-associated pneumonia, diaphragmatic atrophy, pneumothorax, and BPD (5), making timely extubation crucial.

The lack of standardized extubation criteria leads clinicians to rely on ventilator parameters, blood gas analysis, and subjective experience, contributing to high failure rates. Reintubation may cause airway trauma, bradycardia, hypercapnia, and altered cerebral perfusion/oxygenation (6). Identifying objective predictors of extubation failure could reduce reintubations and improve outcomes.

Limited data exist on peri-extubation respiratory management and medication use. Conventional extubation criteria often fail to adequately assess neonatal readiness due to individual variability, and evidence-based predictors remain scarce (7). This study comprehensively analyzes risk factors for extubation failure in NRDS infants to establish predictive indicators and reduce failure rates.

## Methods

### Study population

We enrolled all infants ≤32 weeks' gestation admitted to the NICU of Jiangxi Maternal and Child Health Hospital (1 January 2021–31 May 2024) who were diagnosed with NRDS within 72 h postbirth, received invasive mechanical ventilation, and underwent protocol-based extubation (8).

Groups:

• Success (ES): no reintubation within 72 h post-extubation
• Failure (EF): reintubation required within 72 h

Exclusion criteria:

1. Admission age >3 days or hospitalization <7 days
2. Death/withdrawal pre-extubation
3. Complex congenital heart disease, major malformations, or metabolic disorders
4. Transferred for surgical care
5. Extubation against medical advice
6. Incomplete clinical data

**Figure 1:**
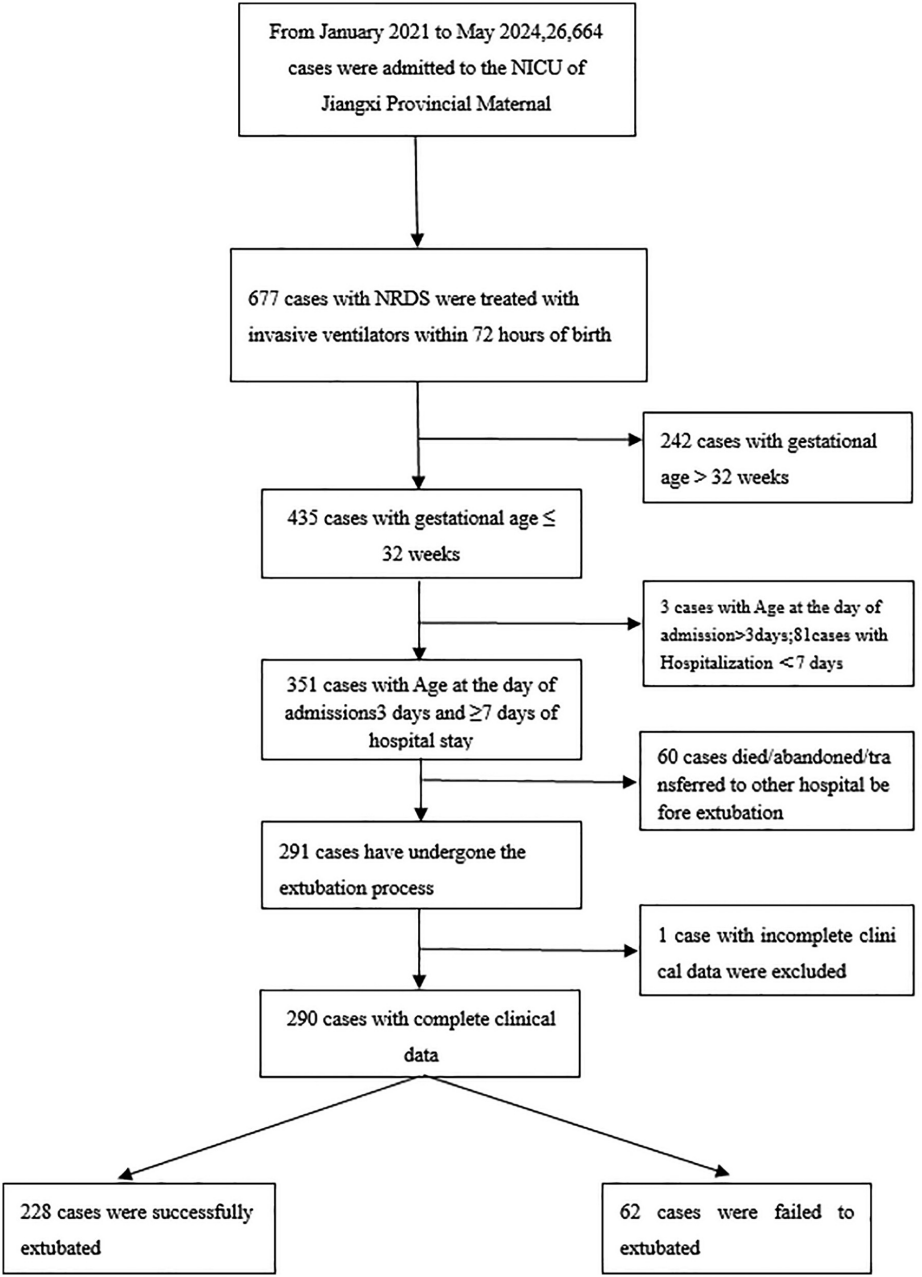
Flow diagram of the study population.

Figure 1 outlines the screening process. Data were extracted from electronic medical records and the NICU database. The study was approved by the hospital's ethics committee (No. EC-KY-2024096, obtained on 18 June 2024).

## Materials and methods

### Definitions

#### Indications for mechanical ventilation

All neonates were ventilated using the Dräger Babylog 8000 ventilator. Mechanical ventilation was initiated according to the 2015 Neonatal Mechanical Ventilation Guidelines (8).

#### Extubation criteria

(1) Weaning process:
  • Initiated when the primary disease improved, infection was controlled, and general condition stabilized with normal blood gas results.
  • Ventilator parameters were gradually reduced (first FiO₂ and PIP, then rate) while monitoring chest movement, SpO₂, and arterial blood gases (ABGs).
(2) Extubation readiness:
  • PIP ≤18 cmH₂O, PEEP 2–4 cmH₂O, rate ≤10 bpm, FiO₂ ≤0.4
  • Normal ABGs (pH, PaCO₂, PaO₂)

#### Extubation failure

Defined as reintubation within 72 h after initial extubation (9).

#### Reintubation Criteria

Required if ≥1 of the following occurred (7):

1. Respiratory dysfunction (hypoxemia, hypercapnia, severe respiratory distress, failure, or massive atelectasis)
2. ABG abnormalities (pH <7.25, PaCO₂ >60 mmHg)
3. Hypoxemia (SpO₂ <88% despite FiO₂ ≥60%)
4. Frequent apnea (>2 episodes/hour or apnea requiring positive pressure ventilation)
5. Hemodynamic instability (major hemorrhage, cardiac arrest)

#### Medication indications

• Intravenous immunoglobulin (IVIG) (10):
  1. Maternal antibody-mediated disorders (e.g., hemolytic disease of the newborn, neonatal thrombocytopenia, neonatal hemochromatosis, myasthenia gravis)
  2. Neonatal infections (bacterial, fungal, or viral)
  3. Immunological disorders (primary immunodeficiency, neonatal Kawasaki disease)
• Azithromycin (11):
  1. Infections (*Ureaplasma urealyticum*, *Mycoplasma*/*Chlamydia*, pertussis)
  2. BPD prophylaxis

### Data collection

Clinical data were collected for all study participants: (1) preterm infant characteristics, including sex, gestational age, birth weight, small for gestational age (SGA), multiple birth, Apgar score, delivery mode, fluid intake in the first 3 days of life, initial ventilator parameters, arterial blood gas analysis, and total hospital stay; (2) perinatal factors, including maternal conditions such as gestational diabetes and hypertensive disorders, antenatal medications (e.g., dexamethasone and insulin), maternal *Ureaplasma urealyticum* (UU) infection, placental abruption, and placenta previa; (3) pre-extubation status, including postnatal age, weight, daily fluid intake, use of intravenous steroids and nebulization, laboratory values within 72 h (white blood cell count, hemoglobin, albumin), arterial blood gases, and ventilator settings; medication administration [including pulmonary surfactant, vasoactive drugs, caffeine, insulin, diuretics, albumin, intravenous immunoglobulin (IVIG), azithromycin, and ibuprofen]; and (4) outcome measures and diagnostic criteria, including (a) retinopathy of prematurity (ROP) diagnosed per ophthalmology consultation following the 2014 Chinese Guidelines for ROP Screening (12); (b) necrotizing enterocolitis (NEC) diagnosed and staged according to the 5th edition of practical neonatology; (c) intraventricular hemorrhage (IVH) classified by Papile grading via cranial ultrasound; (d) bronchopulmonary dysplasia (BPD) severity classified by the maximum respiratory support required at 36 weeks' postmenstrual age or discharge (13); (e) pulmonary hypertension diagnosed by echocardiography per the 2017 Chinese Expert Consensus on Neonatal Pulmonary Hypertension (14).

### Statistical analysis

SPSS 27.0 was used to statistically analyze the data. Measurement data were determined by a one-way Kolmogorov–Smirnov non-parametric test to determine whether they conformed to normal distribution. Measurement data that conformed to normal distribution were expressed as mean ± standard deviation (*x* ± *s*), and independent samples *t*-tests were used for comparison between groups. Measurement data that did not conform to normal distribution were expressed as median (quartile), and Mann–Whitney *U*-tests were used for comparison between groups. Categorical count data were expressed as the number of cases (percentage), and the *χ*^2^ test was used for comparison between groups. *p* < 0.05 was used to indicate that the difference was statistically significant. All statistically different clinical indicators were included in the multiple logistic regression analysis, with *p* < 0.05 indicating a statistically significant difference. Subject work characteristic (ROC) curves were used to evaluate the accuracy of prediction. Calibration curves and the Hosmer–Lemeshow test were used to test conformity and fit. Decision curve analysis (DCA) reflected the net benefit of the model for infants.

## Results

### General information

A total of 435 neonates ≤32 weeks' gestation diagnosed with NRDS within 72 h of birth and receiving invasive mechanical ventilation were admitted to the NICU of Jiangxi Maternal and Child Health Hospital between 1 January 2021 and 31 May 2024. After excluding three cases with admission age >3 days, 81 cases with hospitalization <7 days, and 60 cases of pre-extubation death or treatment withdrawal, 290 cases were included. Based on reintubation requirement within 72 h post-first extubation, infants were categorized into the successful extubation group (ES, *n* = 228) and failed extubation group (EF, *n* = 62), yielding an extubation failure rate of 21.4%. The cohort had a mean gestational age of 28.53 ± 1.82 weeks, median birth weight of 1.10 (0.92, 1.39) kg, 178 males (61.38%), and 109 vaginal deliveries (37.59%).

### Unifactorial intergroup difference analysis of extubation failure

The EF group exhibited significantly lower gestational age, birth weight, pre-extubation weight, and initial serum albumin levels (*p* < 0.05) but higher Day 1 fluid intake, pre-extubation FiO_2_, incidence of hemodynamically significant patent ductus arteriosus (PDA) (>1.5 mm), and severe IVH (*p* < 0.05). Maternal *Ureaplasma urealyticum* (UU) infection and placental abruption were more frequent in the EF group (*p* < 0.05). No significant differences (*p* > 0.05) were observed in sex, Apgar scores, antenatal steroid use, or administration of pulmonary surfactant, caffeine, diuretics, vasoactive drugs, albumin, or IVIG, nor in pre-/post-extubation blood gases or ventilator modes (Tables 1–3).

**Table 1:** Comparison of clinical data of preterm infants in the ES group and EF group.

Variables	Total (*n* = 290)	Extubation success (*n* = 228)	Extubation failure (*n* = 62)	Statistics	*p*
GA, weeks	28.53 ± 1.82	28.77 ± 1.75	27.64 ± 1.82	*t* = 4.48	<0.001
Birth weight, kg	1.10 (0.92, 1.39)	1.20 (0.96, 1.40)	0.94 (0.80, 1.23)	*Z* = −4.00	<0.001
Male, *n* (%)	178 (61.38)	142 (62.28)	36 (58.06)	*χ*^2^ = 0.37	0.545
Multiple births, *n* (%)	108 (37.24)	84 (36.84)	24 (38.71)	*χ*^2^ = 0.07	0.787
Vaginal delivery, *n* (%)	109 (37.59)	82 (35.96)	27 (43.55)	*χ*^2^ = 1.19	0.274
SGA, *n* (%)	24 (8.28)	21 (9.21)	3 (4.84)	*χ*^2^ = 1.23	0.268
Apgar-1 min	6 (5, 8)	6.00 (5, 8)	7.00 (5, 8)	*Z* = −0.25	0.802
Apgar-5 min	9 (8, 9)	9.00 (8, 9)	8.50 (8, 9)	*Z* = −0.46	0.643
D1 fluid intake (ml/kg)	76.17 ± 8.70	75.56 ± 8.03	78.39 ± 10.59	*t* = −2.28	0.023
D2 fluid intake (ml/kg)	89.72 ± 11.47	89.06 ± 11.18	92.16 ± 12.28	*t* = −1.89	0.059
D3 fluid intake (ml/kg)	107.06 ± 12.90	106.34 ± 12.10	109.71 ± 15.31	*t* = −1.83	0.068
First blood Alb	29.10 (27.10, 31.37)	29.30 (27.37, 31.60)	28.80 (26.42, 30.50)	*Z* = −2.19	0.028
Duration time of MV this time (days)	4 (2, 9)	4 (2, 9)	5 (2, 9)	*Z* = −0.20	0.843
The first MV is in normal frequency mode, *n* (%)	149 (51.38)	113 (49.56)	36 (58.06)	*χ*^2^ = 1.41	0.235
Chest x-ray before intubation NRDS ≥ Grade 3, *n* (%)	116 (40.00)	85 (37.28)	31 (50.00)	*χ*^2^ = 3.29	0.070
Prenatal glucocorticoids, *n* (%)	247 (85.17)	195 (85.53)	52 (83.87)	*χ*^2^ = 0.11	0.745
Gestational hypertension, *n* (%)	43 (14.83)	31 (13.60)	12 (19.35)	*χ*^2^ = 1.28	0.258
Gestational diabetes, *n* (%)	57 (19.66)	43 (18.86)	14 (22.58)	*χ*^2^ = 0.43	0.513
Prenatal use of insulin, *n* (%)	20 (6.90)	14 (6.14)	6 (9.68)	-	0.121
Placenta previa, *n* (%)	33 (11.38)	23 (10.09)	10 (16.13)	*χ*^2^ = 1.76	0.184
Placental abruption, *n* (%)	35 (12.07)	22 (9.65)	13 (20.97)	*χ*^2^ = 5.88	0.015
UU infection in the mother, *n* (%)	17 (5.86)	11 (4.82)	6 (9.68)	*χ*^2^ = 17.52	<0.001

SGA, infants born small for gestational age were defined as having birth weight below the 10th percentile; MV, mechanical ventilation; Alb, albumin; NRDS, neonatal respiratory distress syndrome; UU, *Ureaplasma urealyticum*.

**Table 2:** Comparison of data before and after extubation.

Variables	Total (*n* = 290)	Extubation success (*n* = 228)	Extubation failure (*n* = 62)	Statistics	*p*
Pre-extubation					
Day of life	5 (3, 10)	5 (3, 10)	5 (3, 9)	*Z* = −0.30	0.764
Liquid intake (ml/kg)	130 (111, 150)	130 (110, 150)	140 (120, 150)	*Z* = −1.90	0.057
Intravenous steroid, *n* (%)	66 (22.76)	49 (21.49)	17 (27.42)	*χ*^2^ = 0.97	0.324
Budesonide, *n* (%)	70 (24.14)	52 (22.81)	18 (29.03)	*χ*^2^ = 1.03	0.310
WBC within 72 h	11.70 (7.68, 17.09)	11.25 (7.47, 16.98)	13.18 (8.52, 18.09)	*Z* = −1.42	0.154
Hb within 72 h	148 (133, 164)	148 (133, 165)	147 (126, 160)	*Z* = −1.11	0.266
Alb within 72 h	29.40 (26.90, 32.00)	29.80 (27.65, 32.05)	28.75 (26.05, 30.98)	*Z* = −1.77	0.077
Weight (kg)	1.17 (0.95, 1.41)	1.21 (0.97, 1.45)	0.98 (0.83, 1.25)	*Z* = −4.36	<0.001
UU infection	72 (24.83)	58 (25.44)	14 (22.58)	*χ*^2^ = 0.21	0.644
pH	7.38 (7.33, 7.44)	7.38 (7.33, 7.45)	7.36 (7.31, 7.44)	*Z* = −1.16	0.245
PaCO_2_	39.30 (32.10, 45.77)	38.85 (32.05, 44.50)	41.95 (34.83, 47.58)	*Z* = −1.38	0.167
PaO_2_	74.00 (55.50, 94.40)	74.00 (56.65, 94.40)	72.60 (46.33, 95.17)	*Z* = −0.71	0.478
FiO_2_	27 (25, 30)	25 (25, 30)	30 (25, 30)	*Z* = −2.97	0.003
PDA > 1.5 mm, *n* (%)	167 (57.59)	123 (53.95)	44 (70.97)	*χ*^2^ = 5.78	0.016
Severe IVH, *n* (%)	33 (11.38)	18 (7.89)	15 (24.19)	*χ*^2^ = 12.84	<0.001
The initial ventilatory support was CMV, *n* (%)	236 (81.38)	187 (82.02)	49 (79.03)	*χ*^2^ = 0.29	0.592
After extubation Mode of NIV, *n* (%)				-	0.521
HFNC	1 (0.34)	1 (0.44)	0 (0.00)		
CPAP	2 (0.69)	2 (0.88)	0 (0.00)		
NIPPV	158 (54.48)	128 (56.14)	30 (48.39)		
NHFOV	129 (44.48)	97 (42.54)	32 (51.61)		

WBC, white blood cell count; Hb, hemoglobin; Alb, albumin; PaCO_2_, partial pressure of carbon dioxide; PaO_2_, partial pressure of oxygen; FiO_2_, fraction of inspired oxygen; PDA, patent ductus arteriosus; NIV, non-invasive ventilation; HFNC, high flow oxygen; CPAP, continuous positive airway pressure; NIPPV, nasal intermittent positive pressure ventilation; NHFOV, non-invasive high-frequency oscillatory ventilation.

**Table 3:** Drug use in the 48 h before extubation.

Variables	Total (*n* = 290)	Extubation success (*n* = 228)	Extubation failure (*n* = 62)	Statistics	*p*
Pulmonary surfactant, *n* (%)	96 (33.10)	77 (33.77)	19 (30.65)	*χ*^2^ = 0.22	0.643
Caffeine, *n* (%)	263 (90.69)	206 (90.35)	57 (91.94)	*χ*^2^ = 0.14	0.703
Sedation, *n* (%)	44 (15.17)	37 (16.23)	7 (11.29)	*χ*^2^ = 0.92	0.337
Vasoactive drugs, *n* (%)	88 (30.34)	63 (27.63)	25 (40.32)	*χ*^2^ = 3.71	0.054
Insulin, *n* (%)	7 (2.41)	4 (1.75)	3 (4.84)	*χ*^2^ = 0.88	0.349
Diuretic, *n* (%)	37 (12.76)	29 (12.72)	8 (12.90)	*χ*^2^ = 0.00	0.969
Albumin, *n* (%)	13 (4.48)	11 (4.82)	2 (3.23)	*χ*^2^ = 0.04	0.847
Gamma globulin, *n* (%)	33 (11.38)	23 (10.09)	10 (16.13)	*χ*^2^ = 1.76	0.184
Azithromycin, *n* (%)	38 (13.10)	28 (12.28)	10 (16.13)	*χ*^2^ = 0.63	0.426
Ibuprofen, *n* (%)	30 (10.34)	22 (9.65)	8 (12.90)	*χ*^2^ = 0.56	0.456

### Logistic multifactorial regression analysis of extubation failure

Multivariate logistic regression analysis was performed with the extubation outcome as the dependent variable and the above factors with statistically significant differences as independent variables. The results showed that maternal prenatal UU infection (OR = 3.45, 95% CI: 1.02–11.66), placental abruption (OR = 2.98, 95% CI: 1.24–7.15), low body weight at extubation (OR = 0.03, 95% CI: 0.00–0.35), high FiO_2_ (OR = 1.09, 95% CI: 1.01–1.17), severe intracranial hemorrhage (OR = 3.97, 95% CI: 1.69–9.30), and PDA (>1.5 mm) (OR = 2.13, 95% CI: 1.06–4.27) before extubation were independent risk factors for extubation failure (*P* < 0.05) (Table 4).

**Table 4:** Univariate and multivariate logistic regression analysis of extubation failure.

Variables	Univariate					Multivariate				
	*β*	SE	*Z*	*p*	OR (95% CI)	*β*	SE	*Z*	*p*	OR (95% CI)
GA	−0.36	0.09	−4.20	<0.001	0.70 (0.59–0.82)	−0.10	0.14	−0.71	0.475	0.90 (0.68–1.19)
BW	−2.02	0.53	−3.78	<0.001	0.13 (0.05–0.38)	1.73	1.43	1.21	0.227	5.65 (0.34–93.58)
UU infection in the mother	1.10	0.54	2.05	0.040	3.02 (1.05–8.67)	1.24	0.62	2.00	0.046	3.45 (1.02–11.66)
Placental abruption	0.91	0.38	2.37	0.018	2.48 (1.17–5.28)	1.09	0.45	2.44	0.015	2.98 (1.24–7.15)
First blood Alb	−0.09	0.04	−2.15	0.031	0.91 (0.84–0.99)	−0.05	0.05	−0.94	0.347	0.96 (0.87–1.05)
D1 Liquid intake	0.04	0.02	2.22	0.027	1.04 (1.01–1.07)	0.00	0.02	0.20	0.844	1.00 (0.97–1.04)
FiO_2_ before extubation	0.07	0.03	2.29	0.022	1.07 (1.01–1.13)	0.08	0.04	2.26	0.024	1.09 (1.01–1.17)
Weight before extubation	−2.23	0.54	−4.10	<0.001	0.11 (0.04–0.31)	−3.63	1.32	−2.75	0.006	0.03 (0.00–0.35)
PDA > 1.5 mm before extubation	0.74	0.31	2.37	0.018	2.09 (1.14–3.83)	0.76	0.35	2.13	0.033	2.13 (1.06–4.27)
Severe IVH before extubation	1.31	0.39	3.41	<0.001	3.72 (1.75–7.92)	1.38	0.43	3.17	0.002	3.97 (1.69–9.30)

IVH, intraventricular hemorrhage.

### Assessment of the predictive value of risk factors for extubation failure in predicting extubation

**Figure 2:**
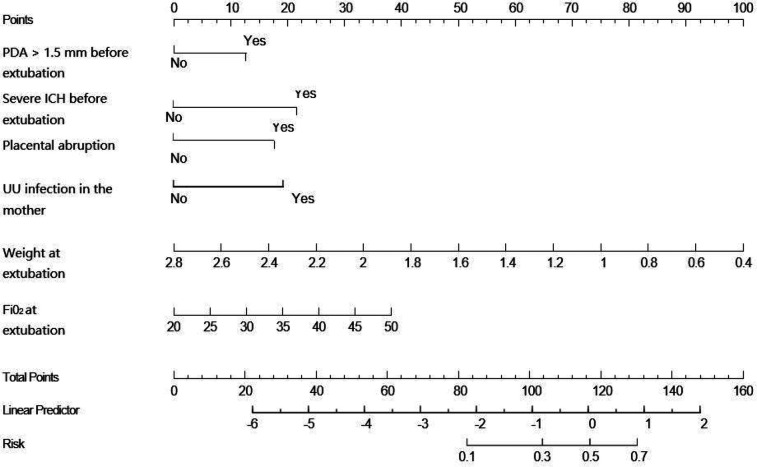
Nomogram of predicted extubation failure in very preterm infants.

**Figure 3:**
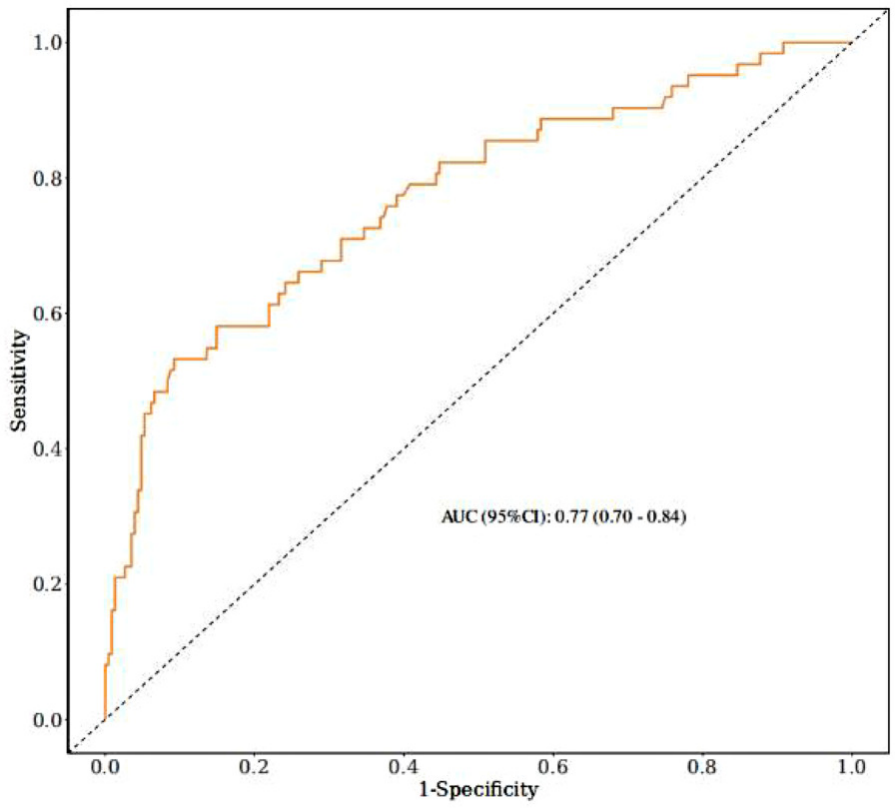
Operating characteristic curve (ROC) and area under the curve (AUC).

**Figure 4:**
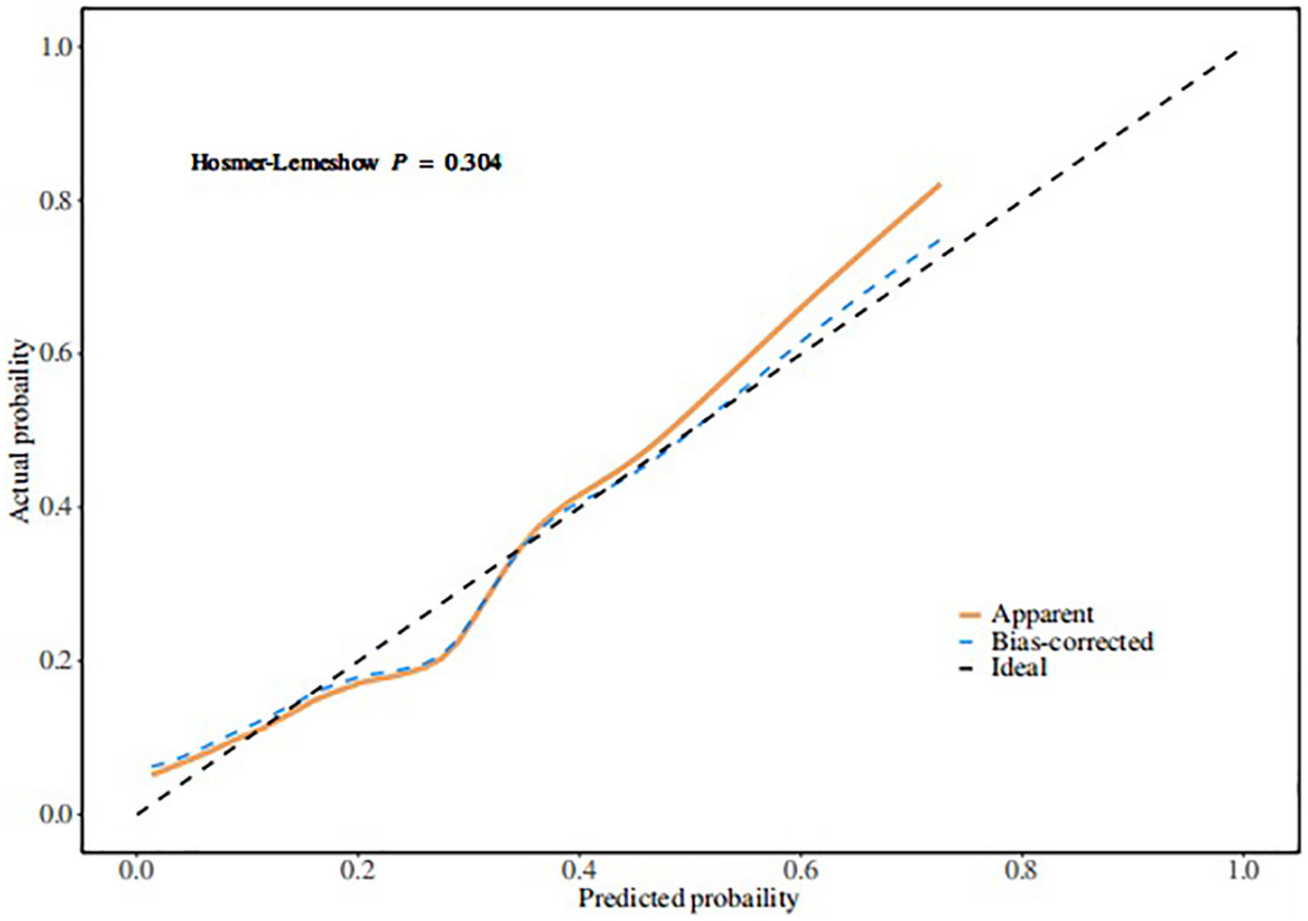
Calibration curve of extubation failure.

**Figure 5:**
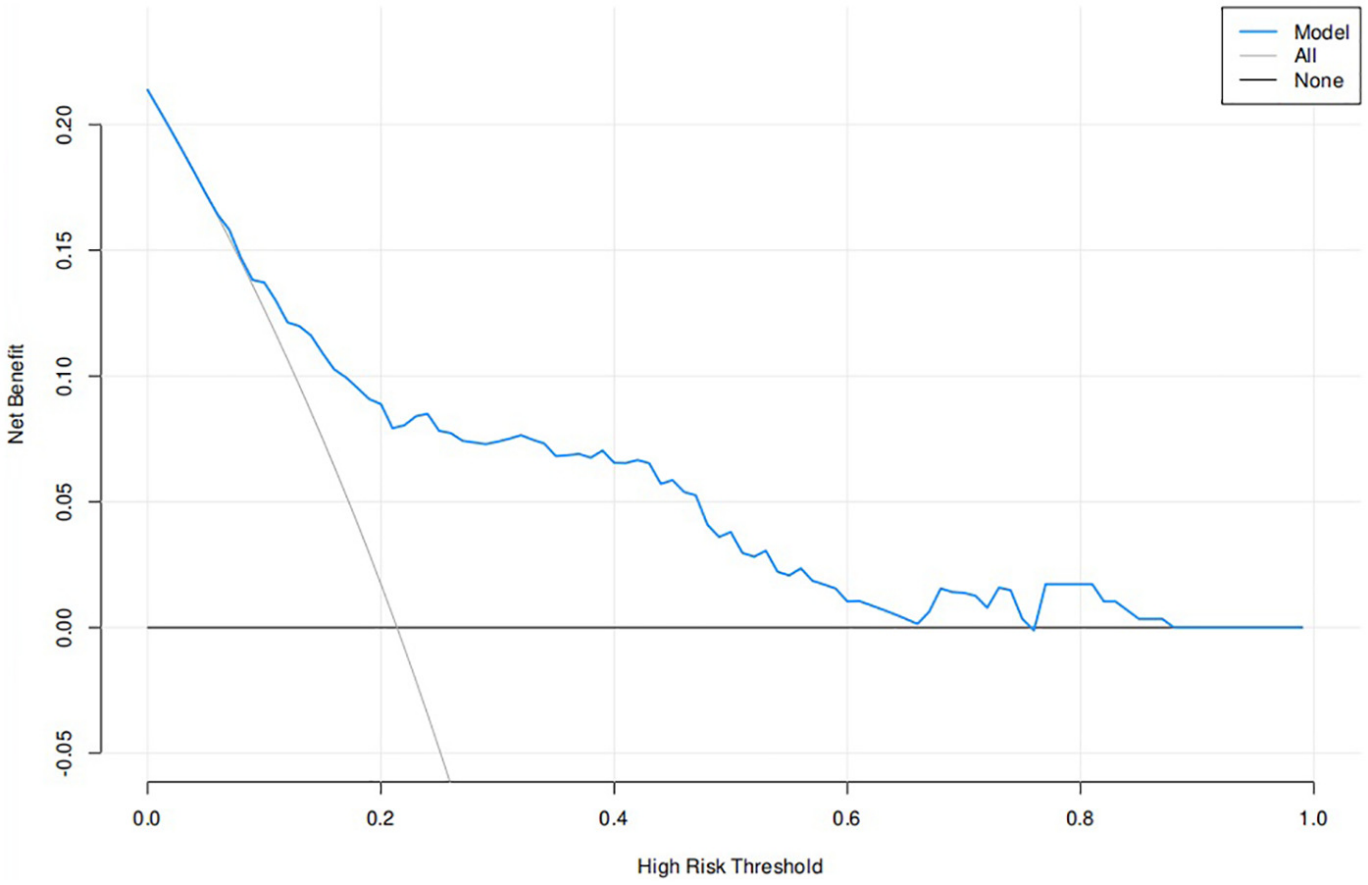
DCA curve of extubation failure.

Based on the multivariate logistic regression model incorporating the six independent risk factors mentioned above, a nomogram for predicting extubation failure was developed using R statistical software (Figure 2). The nomogram quantitatively analyzes each predictor's impact on extubation failure risk, where higher calculated risk scores warrant more cautious extubation timing. The model had an AUC of 0.77 (95% CI: 0.70–0.84), showing moderate predictive power (Figure 3). The Youden index was highest with a sensitivity of 0.91, a specificity of 0.52, a positive predictive value of 0.87, and a negative predictive value of 0.60. Both the Hosmer–Lemeshow test (*p* = 0.304) and the calibration curve evidenced good fit and consistency (Figure 4), and the DCA curves showed a significant net benefit (Figure 5).

## Adverse outcome analysis

The incidence of pulmonary atelectasis (5.26% vs. 24.19%) and BPD (63.60% vs. 79.03%) was higher in the extubation failure group than that in the extubation success group. The duration of the need for invasive mechanical ventilation support during hospitalization [5 (2, 10) vs. 15 (7, 24)] is longer than that in the successful extubation group (*p* < 0.05) (Table 5).

**Table 5:** Comparison of outcomes of preterm infants in the ES group and the EF group.

Variables	Total (*n* = 290)	Extubation success (*n* = 228)	Extubation failure (*n* = 62)	Statistics	*p*
NEC, *n* (%)	27 (9.31)	19 (8.33)	8 (12.90)	*χ*^2^ = 1.21	0.272
Pneumothorax, *n* (%)	7 (2.41)	5 (2.19)	2 (3.23)	*χ*^2^ = 0.00	0.997
Atelectasis, *n* (%)	27 (9.31)	12 (5.26)	15 (24.19)	*χ*^2^ = 20.69	<0.001
Pulmonary hemorrhage, *n* (%)	66 (22.76)	49 (21.49)	17 (27.42)	*χ*^2^ = 0.97	0.324
Pulmonary hypertension, *n* (%)	35 (12.07)	26 (11.40)	9 (14.52)	*χ*^2^ = 0.45	0.505
BPD, *n* (%)				*χ*^2^ = 13.90	0.003
None	96 (33.10)	83 (36.40)	13 (20.97)		
Mild	98 (33.79)	78 (34.21)	20 (32.26)		
Moderate	76 (26.21)	57 (25.00)	19 (30.65)		
Severe	20 (6.90)	10 (4.39)	10 (16.13)		
ROP, *n* (%)				*χ*^2^ = 1.81	0.770
0	170 (58.62)	134 (58.77)	36 (58.06)		
Grade 1	45 (15.52)	36 (15.79)	9 (14.52)		
Grade 2	48 (16.55)	35 (15.35)	13 (20.97)		
Grade 3	26 (8.97)	22 (9.65)	4 (6.45)		
Grade 4	1 (0.34)	1 (0.44)	0 (0.00)		
Duration of MV (days)	7 (3, 13)	5 (2, 10)	15 (7, 24)	*Z* = −6.46	<0.001

NEC, neonatal necrotizing enterocolitis; BPD, neonatal bronchopulmonary dysplasia; ROP, neonatal retinopathy.

## Discussion

There is currently no internationally standardized definition for extubation failure in neonates, with observation windows ranging from 12 h to 7 days post-extubation. A 2023 meta-analysis (15) revealed that commonly used time frames include 48 h (23.5%), 72 h (41.1%), 120 h (8.8%), and 168 h (26.4%) after extubation. Overall, defining extubation failure within a 72 h window captures the majority of clinical failures while avoiding the inclusion of new-onset conditions as causes of extubation failure. An increasing number of studies now define extubation failure as reintubation within 72 h (16). Accordingly, our study adopted this 72 h window to encompass most extubation failures, with a failure rate of 21.4% among infants meeting standard extubation criteria (8). A systematic revision has been conducted to address citation inaccuracies, including: In-text data updates: a 2020 prospective study reported an initial extubation failure rate of 20.8% in neonates, requiring reintubation after failure (17). Research by Kidman demonstrated significantly higher failure rates of 47.1% in extremely preterm infants with gestational age <28 weeks (18). Multiple factors contribute to extubation failure. Independent risk factors identified include lower pre-extubation weight, higher FiO₂, severe IVH, hemodynamically significant patent PDA >1.5 mm, maternal UU infection, and placental abruption. Clinicians should prioritize these factors and implement appropriate interventions to reduce extubation failure rates.

This study identifies maternal placental abruption and UU infection as significant risk factors for extubation failure. Placental abruption compromises fetal oxygen supply *in utero*, with severe cases markedly increasing risks of adverse neonatal outcomes, including fetal growth restriction, stillbirth, preterm delivery, and birth asphyxia (19–21). Existing research (22–24) demonstrates that placental abruption primarily impacts extubation outcomes through its effects on fetal birth weight and gestational age, subsequently predisposing infants to respiratory distress and asphyxia. The resultant prolonged neonatal hypoxia triggers calcium influx, leading to hypocalcemia, which may manifest as edema, seizures, myocardial dysfunction, and coagulopathy—all contributing to significant hypoxic damage to both cerebral and pulmonary tissues (1, 16, 25, 26). This pathophysiological cascade likely underlies the observed difficulties in extubation and prolonged ventilator dependence. However, current evidence predominantly establishes this association indirectly through intermediate outcomes like birth asphyxia, and more direct objective evidence remains to be elucidated through future studies. Concurrently, previous investigations (27, 28) have shown that intrauterine UU infection induces perinatal inflammation and chorioamnionitis. This inflammatory state upregulates cytokine expression, causing pulmonary injury that is further exacerbated by postnatal ventilator-induced volutrauma and increased oxygen demand. In the immature lung, this creates a persistent, dysregulated inflammatory response that impairs pulmonary development, ultimately increasing ventilator dependence and predisposing to extubation failure.

Our study demonstrates that lower pre-extubation weight is significantly associated with higher extubation failure risk. While existing research has primarily focused on gestational age and birth weight analyses, few studies have specifically examined weight at extubation. This finding holds clinical importance as increasing weight reflects concurrent improvements in brain maturity, pulmonary development, and respiratory muscle strength (29). Higher pre-extubation weight indicates more advanced respiratory function and neurological development, consequently reducing the probability of post-extubation respiratory muscle fatigue. The critical role of enhanced brain maturation in respiratory control has been corroborated by other studies (30, 31). Furthermore, our results identify higher FiO₂ at extubation as an independent risk factor for failure. While severe NRDS cases may initially require elevated FiO₂ during mechanical ventilation, clinical management aims to progressively reduce oxygen requirements to the minimal level, maintaining adequate oxygenation. The pre-extubation FiO₂ level serves as an objective indicator of respiratory autonomy. Lower oxygen dependence reflects greater pulmonary maturity and consequently predicts lower reintubation probability, as supported by previous research (1, 25, 30). Elevated FiO₂ requirements typically indicate more pronounced oxygenation deficits, necessitating prolonged ventilatory support and consequently increasing extubation risk.

This study demonstrates that severe IVH prior to extubation serves as a significant risk factor for extubation failure. Mechanical ventilation remains the primary treatment modality for neonates ≤32 weeks' gestation with NRDS. In NRDS infants, fluctuations in pulmonary airflow and alterations in blood oxygen/carbon dioxide levels readily induce cerebral blood flow instability, while endotracheal intubation itself may precipitate hemodynamic changes in premature brains (32). Consequently, mechanically ventilated infants face substantially elevated risks of intracranial hemorrhage, a correlation well-documented in multiple studies (33, 34). Our research identified severe IVH cases through standardized imaging criteria before extubation attempts, with both univariate and multivariate analyses confirming statistical significance. These findings strongly suggest that severe IVH constitutes a major contributor to extubation failure. Although no prior studies have explicitly examined the relationship between IVH and extubation timing, we postulate that severe IVH may compromise central nervous system function, delay neurodevelopment, and induce systemic circulatory instability—all potentially impairing successful extubation. The pathophysiological mechanisms likely involve multiple cerebral insults: Mechanical compression from hematomas causes local hypoxia/ischemia in adjacent tissues, while secondary microglial activation triggers phagocytic activity leading to neuronal necrosis and white matter injury (32). These processes collectively depress respiratory and circulatory function, limiting autonomic regulation post-extubation and ultimately resulting in extubation failure. Further basic and clinical investigations are warranted to elucidate these relationships. Additionally, our study identifies PDA exceeding 1.5 mm as another independent risk factor for extubation failure—a novel association not previously reported. The failure group showed significantly higher prevalence of PDA >1.5 mm at extubation compared with the success group, with multivariate logistic regression confirming this correlation. In preterm infants, persistent PDA stems from poor ductal smooth muscle responsiveness to postnatal oxygen and elevated prostaglandin levels, resulting in increased left-to-right shunting. This hemodynamic alteration elevates pulmonary blood flow, potentially causing pulmonary edema and respiratory deterioration (35), thereby prolonging mechanical ventilation requirements and increasing pulmonary infection risks. Prolonged ventilation further exacerbates the situation through heightened inflammatory mediator release, which suppresses angiogenesis, impairs alveolar development, and worsens pulmonary edema (36). These interconnected factors create a vicious cycle of cardiopulmonary compromise, fostering oxygen dependency and bronchopulmonary dysplasia development that ultimately precipitates extubation failure. Therefore, clinical assessment prior to extubation should carefully evaluate ductal arteriosus status, with appropriate interventions implemented when indicated.

As a retrospective case–control study, this research has several inherent limitations: (1) potential underrepresentation of cases due to unrecorded failed extubation trials during the 1 h pre-extubation observation period routinely conducted in clinical practice; (2) exclusive focus on initial extubation failure despite the clinical reality of multiple reintubations in some infants, warranting future investigation; (3) unavoidable selection and information biases characteristic of single-center designs; and (4) small sample size limiting the ability to conduct internal and external validation, which may have an optimistic bias. Future validation with larger samples is still required. Future research should conduct prospective multicenter cohort studies utilizing standardized data collection systems to comprehensively document the entire weaning process (including the trial withdrawal observation period under ventilator support), thereby improving data completeness and representativeness. Such studies would enable systematic evaluation of the clinical characteristics and risk factors associated with multiple weaning failures in neonates. Additionally, internal and external validation studies should be performed to assess the model's generalizability across different healthcare systems.

In summary, maternal placental abruption and *Ureaplasma urealyticum* infection, combined with low extubation weight, high FiO₂, significant PDA (>1.5 mm), and severe IVH constitute independent risk factors for extubation failure in ≤32-week NRDS infants. For this vulnerable population, extubation timing represents a critical juncture requiring comprehensive evaluation of physiological status, oxygenation parameters, and ductal physiology to guide personalized management. Early intervention targeting modifiable factors (PDA, IVH, infection) may mitigate failure risks and associated adverse outcomes. Extubation failure correlates strongly with atelectasis, BPD development, and prolonged mechanical ventilation. Future multicenter studies should build upon these findings to optimize outcomes in ventilator-dependent neonates.

## Data Availability

The datasets presented in this study can be found in online repositories. The names of the repository/repositories and accession number(s) can be found in the article/Supplementary Material.
